# Clinical Lectures on Diseases of the Ear

**Published:** 1872-02

**Authors:** E. L. Holmes

**Affiliations:** Professor of Ophthalmology and Otology in Rush Medical College, and Surgeon to Chicago Charitable Eye and Ear Infirmary


A MONTHLY RECORD OF

*Medicine, Surgery and the Collateral Sciences.*

EDITED BY J. ADAMS ALLEN, M.D., LL.D.; AND WALTER HAY, M.D.

VOL. XXIX. — FEBRUARY, 1872. — NO. 2.

### Original Communications.

ARTICLE I.—*Clinical Lectures on Diseases of the Ear.* By DR. E. L. HOLMES, Professor of Ophthalmology and Otology in Rush Medical College, and Surgeon to Chicago Charitable Eye and Ear Infirmary.

[Continued from August, 1871.]

EXTERNAL MEATUS—FOREIGN BODIES—IMPACTED CERUMEN—CIRCUMSCRIBED INFLAMMATION.

*Gentlemen :*

By the examination of the external meatus we learn more, in a very great majority of cases, regarding the diagnosis and prognosis of diseases of the ear, than by all other means of investigation.

I can do no better at this time than to urge upon you the very great value of the habit of repeatedly examining the normal external meatus daily in various individuals of different ages. In this way, and in this way alone, will you be able to recognize the peculiarities compatible with health, and distinguish slight abnormal changes. The meatus varies in size, shape, length and direction in different persons. The membrana tympani, as we shall see hereafter, varies in several respects in different healthy individuals.

The external meatus throughout about two-thirds of its length is surrounded with bone. The external third of the canal is surrounded with cartilage; the passage throughout its whole extent is lined with very thin periosteum—perichondrium—and dermoid tissue. The epidermis is so thin and delicate, that it seems to have been regarded by the older authorities as a mucous membrane. To the cartilaginous portion are principally confined the sebaceous and ceruminous follicles and hairs, the latter varying to a remarkable degree in different individuals as regards their number and size. In some aged patients it is almost impossible to gain a good view of the meatus without cutting away these hairs.

In confirmation of these remarks you have each but to look carefully into the meatus of these three persons in whom the ears are perfectly normal.

The examination can generally best be conducted in the manner you see me adopt, which is described in our best text books. The concave reflector concentrates the light and throws it through the conical tube (speculum) into the meatus, and brilliantly illuminates the cavity. A very little practice, either with sunlight, artificial light, or the light from a white cloud, will give you perfect control of the illumination.

Of the abnormal conditions of the external meatus, which the student can generally learn to diagnosticate without difficulty, and which practitioners in the country should be perfectly competent to treat, the following may be enumerated:

1. The presence of foreign bodies.
2. Impacted accumulations.
3. Circumscribed inflammation.
4. Diffused inflammation.

1. The young patient before you gives me an opportunity of showing how foreign bodies can almost always be removed. The boy foolishly crowded a short but quite large piece of slate pencil into the ear. It seems to be held by the cerumen so firmly that it cannot be shaken out. As you inspect the meatus you perceive how difficult, if not impossible, it would be to seize the pencil with any instrument. You have observed how readily it was removed by a little warm water thrown into the meatus.

In regard to this class of cases, the most important rule I can impress on your minds, is, not to do too much. It is seldom they cannot be relieved by throwing warm water vigorously yet not too violently into the ear with a well-made syringe holding two or more ounces.

Irregular or large bodies may become so impacted, and in the efforts of the patient or friends to remove them may become so firmly crowded against the membrana tympani, as to require very great skill and delicacy in the use of instruments to extract them. A bean, for instance, may become so swollen from the warm water introduced with an imperfect syringe as to render its removal less easy. A sharp hook passed flatwise between the side of the meatus and such a foreign body, and then so rotated that the point is engaged in the softened substance, will remove it piece by piece without injury to the ear.

Your ingenuity will occasionally be taxed to remove safely round beads, stones, or other hard substances which are sometimes firmly fixed in the meatus. In such cases bear always in mind the delicacy of the structures behind.

2. The patient before you brings our attention to accumulations in the external meatus. You can see by inspection, with the aid of the speculum and reflector, that the meatus is totally closed, the accumulation extending almost to the external orifice of the meatus. You perceive how readily a few syringefuls of warm water in this case have removed the large mass. It is surprising what enormous collections are often found in the ears, and equally surprising how little they disturb hearing, provided there is the slightest passage past them for the waves of sound. Patients with ears in such condition often suddenly experience almost total loss of hearing on attempting to cleanse the ears with warm water. The accumulations absorb moisture, which causes them to swell and totally fill the meatus. You will sometimes meet obstructions of the meatus, composed almost wholly of soft cerumen. These are most readily removed. In other cases they are composed of small portions of cerumen, mingled with collections of dust and especially with layers of cuticle, which have been thrown off, one after the other, by the irritation caused by the continued contact of these foreign substances. Not unfrequently a very large number of hairs are mingled in the mass, which is, as it were, anchored to the walls of the meatus by the hairs still growing there.

Whenever these masses have been suffered to remain so long as to produce inflammation of the walls of the meatus and of the membrana tympani, you may find their removal by no means an easy task. The slightest touch causes excruciating pain. Even warm water thrown in gently is almost intolerable. It then becomes necessary to soften the concretion by frequently instilling warm water and gradually extracting it piece by piece, with the aid of a small spoon, or forceps, in addition to the syringe. I should remind you that the use of the syringe in the ear for any purpose sometimes produces an unpleasant dizziness, which, however, soon passes away. This vertigo is perhaps specially noticed where there is perforation of the membrana tympani.

3. The symptoms of circumscribed inflammation of the external meatus are, pain, swelling, great tenderness, and a sense of fullness. The hearing is seldom impaired, unless the meatus has become totally closed, or the inflammation has extended to the membrana tympani. The disease is identical with the common furuncle or boil. The diagnosis is usually easily made. Simple inspection reveals the circumscribed swelling. A probe delicately applied to the elevation, will, by the pain produced, show you the central point of inflammation. I presented a case of this kind a few days ago, and showed you the manner in which such swellings should be lanced. I need scarcely dwell upon the fact that in ordinary cases a free incision through the afflicted portion of the meatus to the bone, is the only reliable means of relieving the pain. Warm fomentations should also be applied over the ear till the severe symptoms have disappeared.

It is advisable to inform patients that these furuncles, as in other portions of the body, are liable to appear in series—one no sooner being relieved than another appears. Where there is evident tendency to a recurrence of this form of inflammation, I am confident you will often do good by administering quite large and frequent doses of muriated tincture of iron.

